# Antimicrobial Susceptibility Patterns of Environmental Streptococci Recovered from Bovine Milk Samples in the Maritime Provinces of Canada

**DOI:** 10.3389/fvets.2016.00079

**Published:** 2016-09-15

**Authors:** Marguerite Cameron, Matthew Saab, Luke Heider, J Trenton McClure, Juan Carlos Rodriguez-Lecompte, Javier Sanchez

**Affiliations:** ^1^Department of Health Management, University of Prince Edward Island, Charlottetown, PE, Canada; ^2^Diagnostic Services, Atlantic Veterinary College, University of Prince Edward Island, Charlottetown, PE, Canada; ^3^Department of Pathology and Microbiology, University of Prince Edward Island, Charlottetown, PE, Canada

**Keywords:** *Streptococcus uberis*, *Streptococcus dysgalactiae*, environmental streptococci, antimicrobial susceptibility, bovine, intramammary infection

## Abstract

Determination of antimicrobial susceptibility of bovine mastitis pathogens is important for guiding antimicrobial treatment decisions and for the detection of emerging resistance. Environmental streptococci are ubiquitous in the farm environment and are a frequent cause of mastitis in dairy cows. The aim of the study was to determine patterns of antimicrobial susceptibility among species of environmental streptococci isolated from dairy cows in the Maritime Provinces of Canada. The collection consisted of 192 isolates identified in milk samples collected from 177 cows originating from 18 dairy herds. Results were aggregated into: (1) *Streptococcus uberis* (*n* = 70), (2) *Streptococcus dysgalactiae* (*n* = 28), (3) other *Streptococci* spp. (*n* = 35), (4), *Lactococcus* spp. (*n* = 32), and (5) *Enterococcus* spp. (*n* = 27). Minimum inhibitory concentrations (MICs) were determined using the Sensititre microdilution system and mastitis plate format. Multilevel logistic regression models were used to analyze the data, with antimicrobial susceptibility as the outcome. The proportion of susceptible *S. uberis* ranged from 23% (for penicillin) to 99% (for penicillin/novobiocin), with a median of 82%. All *S. dysgalactiae* were susceptible to all antimicrobials except for penicillin (93% susceptible) and tetracycline (18% susceptible). The range of susceptibility for other *Streptococcus* spp. was 43% (for tetracycline) to 100%, with a median percent susceptibility of 92%. *Lactococcus* spp. isolates displayed percent susceptibilities ranging from 0% (for penicillin) to 97% (for erythromycin), median 75%. For the antimicrobials tested, the minimum inhibitory concentrations were higher for *Enterococcus* spp. than for the other species. According to the multilevel models, there was a significant interaction between antimicrobial and bacterial species, indicating that susceptibility against a particular antimicrobial varied among the species of environmental streptococci and vice versa. Generally, susceptibility decreased with increasing within-herd average somatic cell count, isolates recovered in mid-lactation were more susceptible than isolates recovered in early lactation, and isolates recovered in samples collected post-clinical mastitis were more susceptible than isolates recovered from non-clinical lactating quarters. The results of this research support continued susceptibility of environmental streptococci to beta-lactam antimicrobials. A departure from the expected susceptibility to beta-lactams was the apparent reduced susceptibility of *S. uberis* to penicillin.

## Introduction

Mastitis in dairy cows is a prevalent and costly disease for the dairy industry (1). Treatment and prevention of mastitis represents the most common reason for antimicrobial use on dairy farms (2, 3). Determination of antimicrobial susceptibility patterns of bovine mastitis pathogens is important for guiding antimicrobial treatment decisions and for the early detection of emerging antimicrobial resistance (AMR) among different bacterial species. The role of livestock as a potential source of AMR bacteria or resistance genes is a growing concern (4); therefore, surveillance of AMR in food-producing animals is important from both animal health and human health perspectives.

Environmental streptococci, including *Streptococcus uberis* (*S. uberis*), *Streptococcus dysgalactiae* (*S. dysgalactiae*), and *Enterococcus* spp., are ubiquitous in the farm environment and are a frequent cause of both clinical and subclinical mastitis in dairy cows (5–7). While traditionally classified as environmental pathogens, with the source of the causative pathogen being the environment in which the cow lives, both *S. uberis* and *S. dysgalactiae* have been shown to be occasional agents of contagious mastitis, transmitted from cow-to-cow *via* the milking process (8, 9). In large part, previous reports on the antimicrobial susceptibility of environmental streptococci (most commonly *S. uberis* and *S. dysgalactiae*) do not indicate widespread emerging resistance, despite a long history of exposure to antimicrobial treatments (10–12). However, also present within the literature are occasional reports of reduced susceptibility of *S. uberis* to penicillin (13–15). Considering that penicillin and other beta-lactams are considered to be the first-line of defense against streptococci, as well as the potential for transfer of resistance determinants between species (16), continued surveillance for AMR among field isolates of *S. uberis* and other environmental streptococci is warranted.

A lesser known genus among the large group of environmental streptococci bacteria are the *Lactococcus* spp. Historically, traditional biochemical identification methods have been unreliable for *Lactococcus* spp. and as a result, their importance in bovine mastitis is not well-understood (7). Similarly, reports of the antimicrobial susceptibility profiles of *Lactococcus* spp. are sparse. Matrix-Assisted Laser Desorption/Ionization Time-of-Flight Mass Spectrometry (MALDI-ToF MS) is a powerful instrument capable of accurately identifying thousands of bacteria, including *Lactococcus* spp. (17, 18). This diagnostic tool generates protein spectral fingerprints of unknown samples and compares them to a database of reference spectra in order to derive identification. Application of advanced diagnostic methodologies, such as MALDI-ToF MS, on a routine basis, will help to expand our understanding of clinical importance of *Lactococcus* spp.

While recent investigations have described the AMR profiles of *Staphylococcus aureus* (*S. aureus*), *Klebsiella* spp., and *Escherichia coli* (*E. coli*) isolated on Canadian dairy farms (19), the susceptibility profiles of environmental streptococci associated with bovine intramammary infection (IMI) in Canada have not been well defined. The aim of the current study was to determine the patterns of antimicrobial susceptibility among different species of environmental streptococci isolated in milk samples collected from dairy cows in the Maritime Provinces of Canada.

## Materials and Methods

### Source of Isolates

Isolates for this study were obtained from the Mastitis Pathogen Culture Collection (MPCC) of the Canadian Bovine Mastitis and Milk Quality Research Network (CBMQRN). Isolates within the MPCC were recovered in milk samples collected during a 2-year (2007 and 2008) longitudinal cohort study involving 91 commercial dairy herds from six Canadian provinces. Details of herd selection and sample collection for the cohort study have been described by Reyher et al. (5). Regarding milk sample collection, three sampling series were targeted within the cohort study: (1) longitudinally collected lactational samples from healthy cows (lactational series), (2) samples collected from quarters with clinical mastitis on the day of diagnosis and repeated at 2–3 weeks and 4–5 weeks post-diagnosis (clinical series), and (3) samples collected from cows before the end of lactation and post-calving (dry period series). For lactational and dry period series, both quarter and composite samples were collected; for the clinical series, only the affected quarter was sampled.

### Selection of Isolates

The isolates selected for the current study were identified as *S. uberis, S. dysgalactiae*, or other *Streptococci* species within the MPCC and all isolates originated from herds located in the Maritime Provinces. According to the CBMQRN cohort study database, a total of 241 *S. uberis*, 107 *S. dysgalactiae*, and 377 other *Streptococci* spp. were available. Budgetary limits dictated that approximately 300 isolates could be examined for antimicrobial susceptibility. Isolates were selected to target a maximum of one species type (*S. uberis, S. dysgalactiae*, or other *Streptococci* species) per quarter (or composite sample) per sampling series (lactational, clinical, or dry period). The final collection of isolates comprised 75 *S. uberis*, 36 *S. dysgalactiae*, and 185 other *Streptococci* species previously identified in 296 milk samples collected from 243 cows originating from 18 dairy herds. Over half of the isolates (147/296) were recovered in lactational series samples, 63 were isolated 2–4 weeks after a documented case of clinical mastitis, and 86 were identified in dry period samples (28 at dry off; 58 post-calving).

### Antimicrobial Use

In an associated study involving 17 out of the 18 farms over the same time period, antimicrobial use was estimated using a garbage can audit method (3). With regards to intramammary treatments, a penicillin-based therapy was used most frequently in the treatment of clinical mastitis (94% of the herds), followed by a first generation cephalosporin (cephapirin). All 17 herds reported using intramammary antimicrobial treatment at the end of lactation, the majority of which consisted of penicillin-based products (94% of the herds). With regards to systemic treatments, the most commonly used class were third generation cephalosporins, followed by penicillins [both used in 94% of the herds; Ref. (3)].

### Bacteriology

The isolates were transferred from the MPCC (Université de Montréal, Saint-Hyacinthe, QC, Canada) to the Atlantic Veterinary College (Charlottetown, PE, Canada) in lyophilized form. To re-vive the isolates, 1.0 mL of tryptic soy broth was added to each lyophilized culture. The inoculum was applied to a half plate of blood agar using a sterile swab and was streaked using a sterile disposable loop. Plates were incubated for 18–24 h at 35°C and observed for pure growth. Single colonies were sub-cultured onto blood agar and incubated for 18–24 h at 35°C.

### MALDI-ToF Mass Spectrometry Identification

All isolates were characterized using the Bruker microflex MALDI-ToF MS system and Biotyper 3.0 software (Bruker Daltonik GmbH, Bremen, Germany). Samples were analyzed in duplicate using the direct transfer method as follows: bacterial cells from a single colony were transferred onto a stainless steel target, the spot was air dried at ambient temperature, was overlaid with 1 μl matrix HCCA (α-cyano-4-hydroxy-cinnamic acid) diluted in a solution of 50% acetonitrile and 2.5% trifluoroacetic acid, and allowed to air dry a second time at ambient temperature. The target plate was subsequently introduced into the microflex MALDI-ToF mass spectrometer for automated measurement of mass spectra and comparison to the reference database (Biotyper v3.0). Identifications scores ≥2.0 were required for confident species identification; scores <2.0 and ≥1.7 were considered confident genus identification; isolates with scores <1.7 were re-analyzed in duplicate, and if they failed to achieve a score ≥1.7 on the second round, they were classified as unidentified.

### Determination of Antimicrobial Susceptibility

Antimicrobial susceptibility was determined using the Sensititre microdilution system and mastitis plate format CMV1AMAF (Trek Diagnostic Systems Inc., Cleveland, OH, USA). The mastitis plate contained twofold serial dilutions of the following antimicrobials: ampicillin (0.125–8 μg/mL), ceftiofur (0.5–4 μg/mL), cephalothin (2– 6 μg/mL), erythromycin (0.25–4 μg/mL), oxacillin (2–4 μg/mL), penicillin (0.125–8 μg/mL), penicillin/novobiocin combination (1/2–8/16 μg/mL), pirlimycin (0.5–4 μg/mL), sulfadimethoxine (32–256 μg/mL), and tetracycline (1–8 μg/mL). Pure subcultures were used to make bacterial suspensions that were standardized to a 0.5 McFarland turbidity standard as per manufacturer instructions. For *Streptococci* spp., 100 μL of suspension was delivered to each well; for species other than *Streptococcus* genus, 50 μL was dispensed (20). Subcultures were taken from the suspensions and plated on Columbia agar with 5% sheep blood to confirm purity. The mastitis plates were incubated aerobically at 35°C for 18–24 h (20–24 h for *Streptococci* spp.) and were subsequently read automatically using the Sensititre ARIS^®^ (Trek Diagnostic Systems Inc., Cleveland, OH, USA). In case of an error message, the plates were read manually using the Sensititre Vizion System^®^ (Trek Diagnostic Systems Inc., Cleveland, OH, USA). Quality control in accordance with the Clinical Laboratory and Standards Institute (CLSI) using *S. aureus* ATCC 29213 and *E. coli* ATCC 25922 was performed for each new lot of plates (20).

*Aerococcus viridans* is generally considered apathogenic (21); therefore, only *Aerococcus* spp. isolates recovered in samples that had an somatic cell count (SCC) > 200,000 cells/mL were subjected to minimum inhibitory concentration (MIC) determination. Also, omitted from susceptibility testing were bacterial isolates other than streptococci and isolates without MALDI-ToF identification. When the same bacterial species was recovered more than once in the same cow, only one isolate per cow was considered. Isolates originating from post-mastitis samples were preferentially chosen; otherwise, the single isolate within a cow was chosen by random selection using Microsoft Excel’s (Excel 2010, Microsoft Corporation) random number generator.

Isolates identified as resistant to erythromycin and sensitive to pirlimycin were tested for inducible lincosamide resistance using the *D*-test (22). Briefly, erythromycin (15 μg) disk was placed at a distance of 15 mm (edge to edge) from clindamycin (2 μg) disk on a Mueller–Hinton agar plate, previously inoculated with 0.5 McFarland standard bacterial suspensions. Following overnight incubation at 37°C, flattening of zone (D-shaped) around clindamycin in the area between the two disks, indicated inducible clindamycin resistance.

### Data Analysis

The MIC was defined as the lowest concentration of antibiotic that inhibited bacterial growth. If growth was not inhibited at the highest antimicrobial concentration, the isolate was considered to be inhibited at the next highest twofold dilution. *Enterococcus* spp. are considered intrinsically resistant to cephalosporins, erythromycin, and pirlimycin; therefore, no MICs were reported for these bacteria–antimicrobial combinations. Also excluded were the MICs for oxacillin and sulfadimethoxine. Oxacillin is included in the commercial plate to test for methicillin resistant *S. aureus*, which was not of interest in the current study; sulfadimethoxine is rarely used for the treatment and control of mastitis, and the Sensititre manufacturer’s instructions for interpretation of the sulfadimethoxine MIC state that they must be manually read, which was not done. Using guidelines provided by the CLSI (20, 23), isolates were categorized as susceptible, intermediate, or resistant to each antimicrobial.

### Statistical Analysis

Frequency distributions of MIC, the MIC_50_, and MIC_90_ were calculated for each antimicrobial–species combination. Multilevel logistic regression models with herd and isolate as random effects were used to analyze the data, considering antimicrobial susceptibility as the outcome. Two separate models were constructed, one without *Enterococcus* spp. and one with *Enterococcus* spp. but without the antimicrobials to which *Enterococcus* spp. are considered intrinsically resistant. In the models, the event of interest (antimicrobial susceptibility) was coded zero for resistant/intermediate and one for susceptible isolates. The main predictors were antimicrobial type, bacterial species, and an interaction between antimicrobial and bacterial species. Additional independent variables examined included a categorical variable for stage of lactation at the time of sample collection consisting of early [0–100 days in milk (DIM)], mid (101–200 DIM), and late (201+ DIM); parity categorized as 1st, 2nd, and ≥3rd; lactating herd size dichotomized as ≤80 cows and >80 cows; average within-herd SCC (1,000 cells/mL) categorized as low (≤150), moderate (>150 to ≤250), and high (>250); average within-herd 24 h milk yield (in kilogram) dichotomized into ≤31 and >31; average within-herd DIM dichotomized into ≤170 and >170. Within-herd averages were calculated using data collected by the regional Dairy Herd Improvement agency over the course of the study period (2007 and 2008). In the models described above, sample type was considered to be an intervening variable and, therefore, removed from the models. In temporal terms, the quarter became infected with the bacteria prior to sample collection; therefore, sample type would intervene in the pathway between bacterial species and antimicrobial susceptibility. Thus, a third random effects logistic model for sample type was also considered. In this model, sample type was categorized as clinically healthy lactational (including all isolates recovered in lactational and pre-dry-off samples), post-calving (collected between 0 and 14 DIM), and post-clinical mastitis (collected between 2 and 5 weeks after a recorded clinical mastitis event).

In all models, random effects for multiple antimicrobial susceptibilities within isolates and isolates within herds were examined. Considering that very few cows contributed more than one isolate to the dataset (mean = 1.08 isolate/cow; range: 1–3), clustering at the cow level was not considered to have a sizeable effect on the model results. Similarly, only five quarters contributed more than one isolate to the dataset, thus, a random effect for quarter was not included. Pairwise correlation analysis was used to detect collinearity among the predictors. Unconditional associations between the explanatory variables and the outcome were examined using simple univariable multilevel logistic regression, and variables with *P* values ≤0.20 were offered for inclusion in the multivariable models. A backwards stepwise procedure was used to determine the final models, and significance was declared at a *P* value ≤0.05. Excluded potentially confounding variables were reintroduced into the model and were retained if they changed the other model coefficients substantially (>20%). Once the final models were reached, the fit was evaluated by examination of residual plots (24). Intraclass correlation coefficients (ICCs) were calculated using the latent variable approximation to estimate the amount of clustering within herds and within isolates (24). Statistical analyses were performed using Stata IC 13.1 (25).

## Results

### MALDI-ToF Mass Spectrometry Identification

The identification of streptococci isolates based on MALDI-ToF MS are listed in Table 1. Of the 296 isolates, 87.8% (260/296) had identification scores ≥2.0 and secure species identification; secure genus identification (score ≥1.7 to <2.0) was achieved for 5.7% (17/296); no identification (score <1.7) was made for 1.7% (5/296) of the isolates. Difficulties in discrimination between species were reported for 14 isolates (4.7%) that had scores ≥2.0 for two different species of *Streptococci*, thus these isolates could only be identified to the genus level. The four most common species among the collection of other environmental streptococci (i.e., not *S. uberis* or *S. dysgalactiae*) were *A. viridans, Enterococcus faecalis, Lactococcus garvieae*, and *Lactococcus lactis* (Table 1). Two non-streptococci organisms were identified: an *Arcanobacterium pluranimalium* and a *Staphylococcus chromogenes*.

**Table 1:** **Identification of 296 isolates of environmental streptococci recovered from bovine milk samples using matrix-assisted laser desorption/ionization time-of-flight mass spectrometry**.

	Number	% of total
**Species level^a^**		
* Streptococcus uberis*	80	27.0
* Aerococcus viridans*	62	21.0
* Streptococcus dysgalactiae*	34	11.5
* Enterococcus faecalis*	20	6.8
* Lactococcus garvieae*	18	6.1
* Lactococcus lactis*	14	4.7
* Enterococcus saccharolyticus*	8	2.7
* Streptococcus parauberis*	6	2.0
* Streptococcus gallolyticus*	4	1.4
* Enterococcus faecium*	3	1.0
* Enterococcus pseudoavium*	3	1.0
* Streptococcus cristatus*	1	0.3
* Streptococcus parasanguinis*	1	0.3
* Enterococcus avium*	1	0.3
* Enterococcus casseliflavus*	1	0.3
* Enterococcus hirae*	1	0.3
* Enterococcus villorum*	1	0.3
* Arcanobacterium pluranimalium*	1	0.3
* Staphylococcus chromogenes*	1	0.3
**Genus level^b^**		
* Aerococcus* spp.	11	3.7
* Streptococcus* spp.	5	1.7
* Lactococcus* spp.	1	0.3
**Low discrimination**^c^		
* Streptococcus lutetiensis*/*equinis*	7	2.4
* Streptococcus dysgalactiae*/*canis*	4	1.4
* Streptococcus gallolyticus*/*alactolyticus*	1	0.3
* Streptococcus gallolyticus*/*lutetiensis*	1	0.3
* Streptococcus pneumonia*/*mitis*	1	0.3
**No identification^d^**	5	1.7
**Total**	296	100

*^a^Identification score ≥2.0; considered secure species identification*.

*^b^Identification score ≥1.7 and <2.0; considered secure genus identification*.

*^c^Identification score ≥2.0 for two different Streptococci species; therefore, only genus level classification was possible*.

*^d^Identification score <1.7; considered no reliable identification*.

### Antimicrobial Susceptibility

Only 8.2% (6/73) of *Aerococcus* spp. isolates were recovered in samples that had an SCC > 200,000 cells/mL and were subjected to MIC determination. A total of five isolates were excluded from susceptibility testing due to lack of MALDI-ToF identification and two were excluded because they were not environmental streptococci (*A. pluranimalium* and *S. chromogenes*). Of the 222 isolates remaining, nine did not grow on the MIC plate after two attempts and were dropped from the analysis. The remaining 213 isolates with MIC data were recovered from 177 individual cows. Nineteen cows had the same bacterial species identified in two separate samples and one cow had the same species identified in three separate samples. In these cases, only one isolate per species per cow was included in the final analysis. The selected 192 isolates were detected in 186 individual quarters. The number of isolates per herd (*n* = 18) ranged from 2 to 26, with a median of 9. The results of antimicrobial susceptibility testing of the 192 isolates are presented in Table 2. Results were aggregated into five categories: (1) *S. uberis* (*n* = 70), (2) *S. dysgalactiae* (*n* = 28), (3) other *Streptococci* spp. (including *Aerococcus* spp.; *n* = 35), (4) *Lactococcus* spp. (*n* = 32), and (5) *Enterococcus* spp. (*n* = 27).

**Table 2:** **Distribution of minimum inhibitory concentrations (MIC) for *Streptococcus uberis* (*n* = 70), *Streptococcus dysgalactiae* (*n* = 28), *Streptococcus* spp.^a^ (*n* = 35), *Lactococcus* spp.^b^ (*n* = 32), and *Enterococcus* spp.^c^ (*n* = 27) recovered from bovine milk samples**.

		Distribution (%) of MIC (g/mL)													
Antimicrobial	Species	Range	N	%Susc.f	0.06	0.125	0.25	0.5	1	2	4	8	16	MIC_50_^h^	MIC_90_^i^
Ampicillin^d^	*S. uberis*	0.06–4	70	71.4	14.3	5.7	51.4	22.9	0.0	1.4	4.3			0.25	0.5
	*S. dysgalactiae*	0.06–4	28	100.0	92.9	3.6	3.6	0.0	0.0	0.0	0.0			≤0.06	≤0.06
	*Streptococcus* spp.	0.06–4	35	91.5	62.9	22.9	5.7	0.0	8.6	0.0	0.0			≤0.06	0.25
	*Lactococcus* spp.	0.06–4	32	46.9	0.0	0.0	46.9	37.5	9.4	3.1	3.1			0.5	1
	*Enterococcus* spp.	0.125–8	27	100.0		25.9	3.7	33.3	33.3	3.7	0.0	0.0		0.5	1

Ceftiofur^e^	*S. uberis*	0.25–2	70	98.5			21.4	20.0	51.4	5.7	1.4			1	1
	*S. dysgalactiae*	0.25–2	28	100.0			100.0	0.0	0.0	0.0				≤0.25	≤0.25
	*Streptococcus* spp.	0.25–2	35	91.4			65.7	14.3	11.4	0.0	8.6			≤0.25	1
	*Lactococcus* spp.	0.25–2	31	96.8			19.4	38.7	35.5	3.2	3.2			0.5	1

Cephalothin^d^	*S. uberis*	1–8	70	98.6					87.1	8.6	0.0	2.9	1.4	≤1	2
	*S. dysgalactiae*	1–8	28	100.0					100.0	0.0	0.0	0.0		≤1	≤1
	*Streptococcus* spp.	1–8	35	100.0					97.1	2.9	0.0	0.0		≤1	≤1
	*Lactococcus* spp.	1–8	32	81.3					0.0	21.9	37.5	21.9	18.8	4	>8

Erythromycin^d^	*S. uberis*	0.125–2	70	85.7		85.7	0.0	0.0	2.9	4.3	7.1			≤0.125	2
	*S. dysgalactiae*	0.125–2	28	100.0		100.0	0.0	0.0	0.0	0.0				≤0.125	≤0.125
	*Streptococcus* spp.	0.125–2	35	85.8		82.9	2.9	0.0	8.6	0.0	5.7			≤0.125	1
	*Lactococcus* spp.	0.125–2	32	96.9		93.8	3.1	0.0	3.1	0.0				≤0.125	≤0.125

Penicillin^d^	*S. uberis*	0.06–4	70	22.9	14.3	8.6	44.3	24.3	2.9	0.0	2.9	2.9		0.25	0.5
	*S. dysgalactiae*	0.06–4	28	92.9	89.3	3.6	7.1	0.0	0.0	0.0	0.0			≤0.06	0.125
	*Streptococcus* spp.	0.06–4	35	85.7	54.3	31.4	5.7	5.7	2.9	0.0	0.0			≤0.06	0.25
	*Lactococcus* spp.	0.06–4	32	0.0	0.0	0.0	3.1	50.0	40.6	0.0	3.1	3.1		0.5	1
	*Enterococcus* spp.	0.125–8	27	100.0		25.9	0.0	0.0	3.7	63.0	3.7	3.7		2	2

Penicillin/novobiocin^e^	*S. uberis*	0.5/1–4/8	70	98.6				98.6	0.0	0.0	1.4			≤0.5	≤0.5
	*S. dysgalactiae*	0.5/1–4/8	28	100.0				100.0	0.0	0.0	0.0			≤0.5	≤0.5
	*Streptococcus* spp.	0.5/1–4/8	35	100.0				100.0	0.0	0.0	0.0			≤0.5	≤0.5
	*Lactococcus* spp.	0.5/1–4/8	32	93.8				81.3	12.5	0.0	3.1	3.1		≤0.5	1
	*Enterococcus* spp.	1/2–8/16	27	ND^g^					96.3	3.7	0.0	0.0		≤1	≤1

Pirlimycin^e^	*S. uberis*	0.25–2	70	78.6			62.9	0.0	0.0	15.7	21.4			≤0.25	>2
	*S. dysgalactiae*	0.25–2	28	100.0			100.0	0.0	0.0	0.0				≤0.25	≤0.25
	*Streptococcus* spp.	0.25–2	35	100.0			88.6	2.9	5.7	2.9				≤0.25	0.5
	*Lactococcus* spp.	0.25–2	32	40.6			18.8	18.8	3.1	0.0	59.4			>2	>2

Tetracycline^d^	*S. uberis*	0.5–4	70	60.0				60.0	0.0	0.0	1.4	38.6		≤0.5	>4
	*S. dysgalactiae*	0.5–4	28	17.8				3.6	7.1	7.1	28.6	53.6		4	>4
	*Streptococcus* spp.	0.5–4	35	42.9				34.3	5.7	2.9	8.6	48.6		>4	>4
	*Lactococcus* spp.	0.5–4	32	68.8				59.4	9.4	0.0	6.3	25.0		≤0.5	>4
	*Enterococcus* spp.	1–8	27	37.0					37.0	0.0	0.0	0.0	63.0	>8	>8

*Isolates with growth at the highest concentration tested are presented in the next highest concentration*.

*Vertical lines indicate CLSI (20, 23) breakpoints*.

*^a^Streptococcus spp. other than S. uberis, S. dysgalactiae, and S. agalactiae*.

*^b^L. lactis, L. garvieae*.

*^c^E. faecalis, E. saccharolyticus, E. faecium, E. pseudoavium, E. avium, E. casseliflavus, and E. hirae*.

*^d^Interpretive criteria based on human data*.

*^e^Interpretive criteria based on bovine mastitis data*.

*^f^Percent of susceptible isolates according to CLSI (20, 23)*.

*^g^ND, no interpretive criteria was available*.

*^h^The MIC value that inhibits growth of 50% of the isolates*.

*^i^The MIC value that inhibits growth of 90% of the isolates*.

Of all the antimicrobials considered, tetracycline displayed the smallest proportion of susceptible isolates. Greater than 90% of the isolates within each species group were sensitive to ceftiofur and penicillin/novobiocin; >80% of isolates were sensitive to cephalothin and erythromycin (Table 2). There was a wide range of susceptibility among the species groups to ampicillin, penicillin, and pirlimycin.

Generally, *S. dysgalactiae* displayed the most susceptibility to the tested antimicrobials and 100% of the isolates were inhibited at the lowest concentration of ceftiofur, cephalothin, erythromycin, penicillin/novobiocin, and pirlimycin. The range of susceptibility for *S. dysgalactiae* was 17.8% (for tetracycline) to 100%, with a median of 100% (Table 2).

Isolates of *S. uberis* were less susceptible than *S. dysgalactiae* to all antimicrobials except for tetracycline. The proportion of susceptible *S. uberis* isolates ranged from 22.9% (for penicillin) to 98.6% (for penicillin/novobiocin), with a median of 82.2% (Table 2).

Isolates of other *Streptococcus* spp. were generally intermediate between *S. uberis* and *S. dysgalactiae*. All isolates were sensitive to cephalothin, penicillin/novobiocin, and pirlimycin. The range of susceptibility for other *Streptococcus* spp. was 42.9% (for tetracycline) to 100%, with a median percent susceptibility of 91.5% (Table 2). Of the six isolates of *Aerococcus* spp., two were pansusceptible (susceptible to all the tested antimicrobials) and four were resistant to tetracycline (MIC > 4 μg/mL); two also displayed intermediate susceptibility to ceftiofur (MIC > 2 μg/mL), and another was intermediately susceptible to penicillin (MIC = 0.25 μg/mL).

*Lactococcus* spp. isolates were overall the least susceptible to the tested antimicrobials, with percent susceptibilities ranging from 0% (for penicillin) to 96.9% (for erythromycin), median 75.1% (Table 2).

As a result of intrinsic resistance to cephalosporins, erythromycin, and pirlimycin, and lack of breakpoint criteria for penicillin/novobiocin, it is difficult to compare isolates of *Enterococcus* spp. to the other species. For the antimicrobials that were tested, the MIC_50_ and MIC_90_ were higher for *Enterococcus* spp. than for any of the other species (Table 2).

Two isolates of *S. uberis* and seven isolates of *Streptococcus* spp. were identified as resistant to erythromycin and sensitive to pirlimycin and were tested for inducible lincosamide resistance using the *D*-test. All isolates were negative.

At the isolate level, the proportion of antimicrobials to which an isolate was considered susceptible ranged from 12.5 to 100%, with a median of 87.5%. At the herd level, the proportion of susceptible isolates across all the antimicrobials ranged from 54.2 to 91.6%, with a median of 81.4%.

### Logistic Regression Models

**Figure 1:**
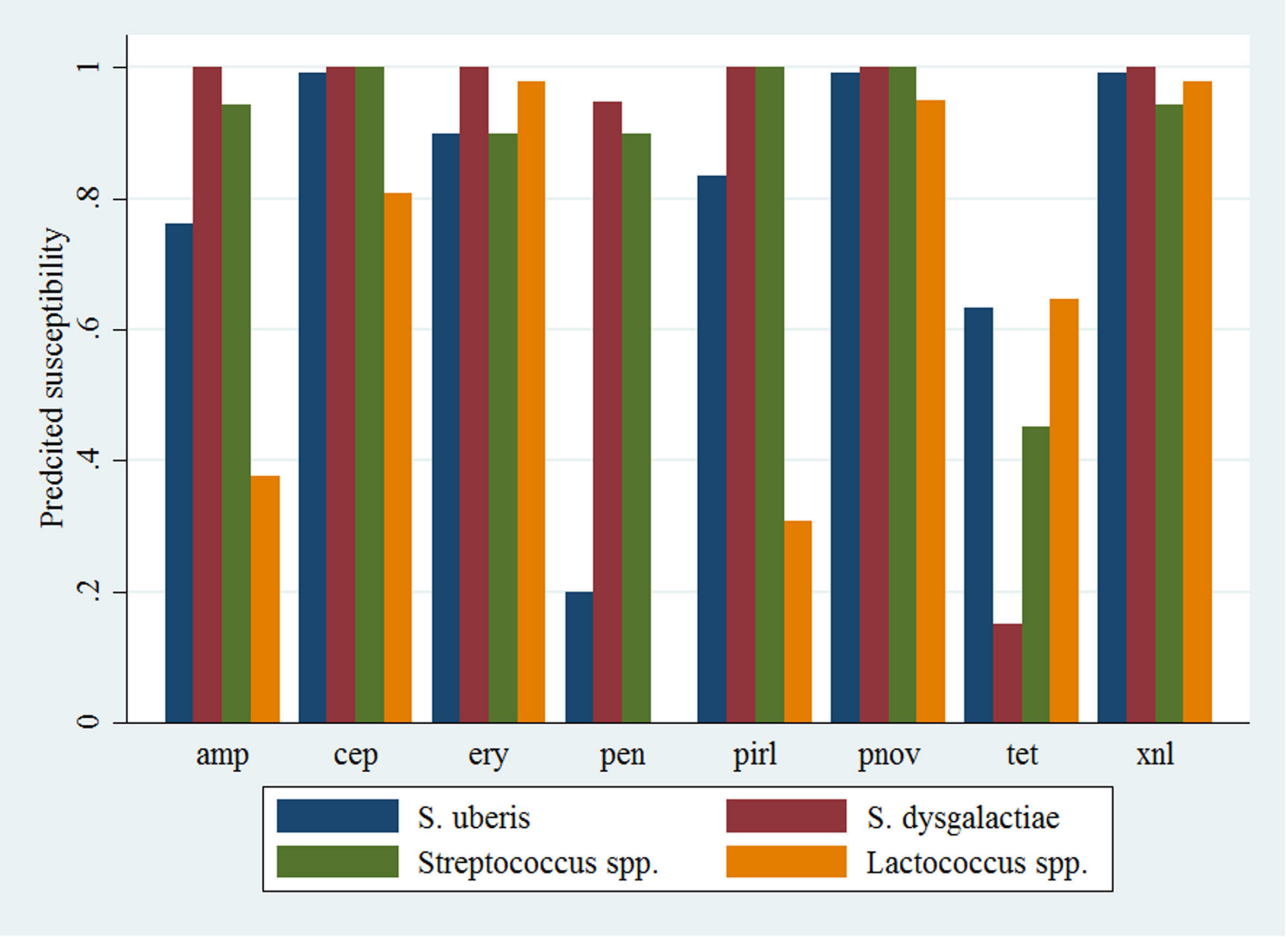
**Predicted probability of antimicrobial susceptibility for isolates of environmental streptococci, excluding *Enterococcus* spp., against eight antimicrobials**. Presented estimates are for isolates recovered from cows in early lactation (0–100 days in milk) and from herds with a low within-herd average somatic cell count (<150,000 cells/mL). amp, ampicillin; xnl, ceftiofur; cep, cephapirin; ery, erythromycin; pen, penicillin; pnov, penicillin + novobiocin; pirl, pirlimycin; tet, tetracycline.

Descriptive data for the cow and herd level variables considered in the models are presented in Table 3. Pairwise correlation analysis did not identify any potential issues with collinearity among the predictor variables. The results of the logistic model of susceptibility of all isolates except *Enterococcus* spp. against eight antimicrobials are presented in Table 4. The interaction between antimicrobial and species was significant, and a graphical display of the relationship is presented in Figure 1. Within-herd average SCC was significantly associated with susceptibility. When compared to a baseline of low SCC (<150,000 cells/mL), isolates recovered in herds with a high SCC were less likely to be susceptible, but isolates from herds with a moderate SCC did not show a significant difference in susceptibility. The odds of susceptibility for isolates recovered in mid-lactation were higher, and the odds for isolates recovered in late lactation were lower when compared with isolates that were identified in early lactation cows. Based on the variance components, clustering at the isolate level was much greater than clustering at the herd level. The correlation among antimicrobial susceptibilities of two isolates from the same herd was lower (ICC = 0.02) than the correlation of the susceptibilities within an isolate (ICC = 0.26).

**Table 3:** **Descriptive statistics for cow and herd level factors considered in the analysis of the susceptibility of bovine intramammary infection-associated environmental streptococci against eight antimicrobials**.

Variable	*N*	%	Minimum	25th percentile	Median	75th percentile	Maximum
**Lactation number**
1	33	18.6	–	–	–	–	–
2	44	24.9	–	–	–	–	–
3	100	56.5	–	–	–	–	–
Stage of lactation at sample collection							
Early (0–100 days in milk)	92	52.0	–	–	–	–	–
Mid (101–200 days in milk)	40	22.6	–	–	–	–	–
Late (201+ days in milk)	45	25.4	–	–	–	–	–
Lactating herd size	–	–	41	50	58	83	199
Mean days in milk^a^	–	–	146	167	174	184	215
Mean 24 h milk production (kg)^a^	–	–	26	29	31	32	40
Mean somatic cell count (1,000 cells/mL)^a^	–	–	107	161	183	266	327

*^a^Within herd averages were calculated using data collected by the regional Dairy Herd Improvement agency over the course of the study period (2007 and 2008)*.

**Table 4:** **Final multilevel logistic regression model of the susceptibility^a^ of 165 environmental streptococci isolates from bovine milk tested against 8 antimicrobials**.

	β	SE(β)	OR	95%CI (OR)		*P* value	Overall *P* value
**Intercept**	1.26	0.48	–	–	–	–	
**Antimicrobial**							
Ampicillin	0.66	0.41	1.94	0.87	4.33	0.107	
Ceftiofur	4.46	1.08	86.51	10.35	723.00	<0.001	
Cephalothin	4.46	1.08	86.51	10.35	723.00	<0.001	
Erythromycin	1.75	0.48	5.74	2.26	14.61	<0.001	
Penicillin	−2.11	0.44	0.12	0.05	0.29	<0.001	
Pen/novobiocin	4.46	1.08	86.51	10.35	723.00	<0.001	
Pirlimycin	1.14	0.43	3.14	1.34	7.34	0.008	
Tetracycline	Ref.	–	–	–	–	–	
**Species**							
*Streptococcus uberis*	Ref.	–	–	–	–	–	
*Streptococcus dysgalactiae*	−2.46	0.67	0.09	0.02	0.32	<0.001	
*Streptococcus* spp.^b^	−0.81	0.54	0.44	0.15	1.27	0.129	
*Lactococcus* spp.^c^	0.06	0.59	1.06	0.33	3.38	0.917	
**Antimicrobial x species**^d^							<0.001
**Herd somatic cell count**^e^							0.002
≤150,000 cells/mL	Ref.	–	–	–	–	–	
151,000–250,000 cells/mL	−0.71	0.42	0.49	0.21	1.12	0.090	
251,000–400,000 cells/mL	−1.21	0.46	0.30	0.12	0.74	0.009	
**Days in milk**^f^							0.034
0–100	Ref.	–	–	–	–	–	
101–200	0.78	0.35	2.19	1.09	4.38	0.027	
201+	−0.69	0.33	0.50	0.26	0.96	0.036	

**Variance**	**Estimate**	**SE**					
Herd level	0.09	0.17					
Isolate level	1.09	0.42					

*^a^Susceptible according to CLSI (20, 23)*.

*^b^Streptococcus spp. other than S. uberis, S. dysgalactiae, and S. agalactiae*.

*^c^L. lactis and L. garvieae*.

*^d^Interaction term between antimicrobial and species*.

*^e^Herd somatic cell count = average within-herd somatic cell count from 2007 to 2008*.

*^f^Days in milk at the time of milk sample collection*.

*OR, odds ratio*.

**Figure 2:**
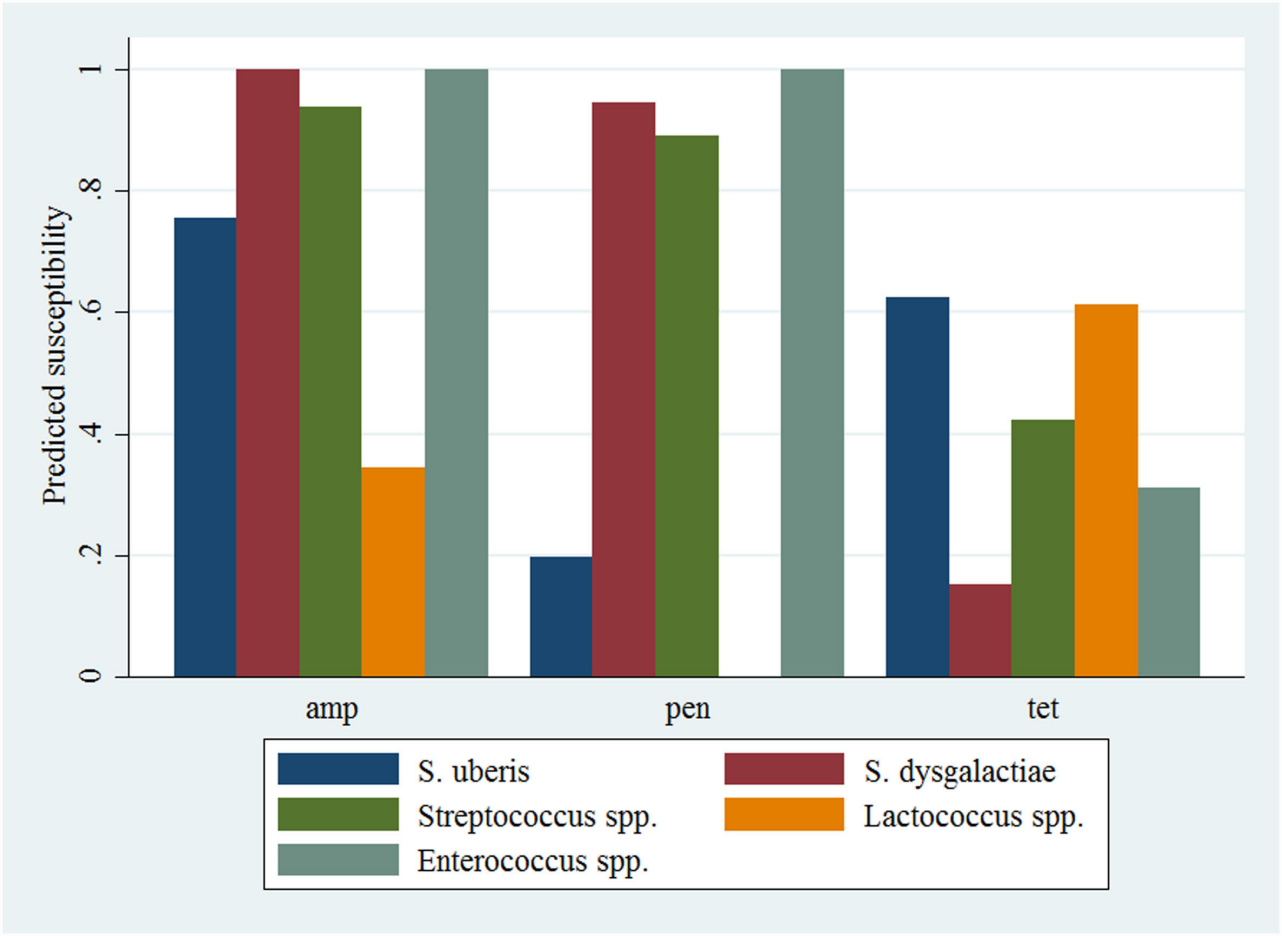
**Predicted probability of antimicrobial susceptibility for isolates of environmental streptococci, including *Enterococcus* spp., against three antimicrobials**. Presented estimates are for isolates recovered from cows in early lactation (0–100 days in milk) and from herds with a low within-herd average somatic cell count (<150,000 cells/mL). amp, ampicillin; pen, penicillin; tet, tetracycline.

The results of the model of all isolates against three antimicrobials are presented in Table 5. The interaction between antimicrobial and species was significant, and a graphical display of the relationship is presented in Figure 2. Within-herd average SCC was significantly associated with susceptibility. When compared to a baseline of low SCC (<150,000 cells/mL), isolates recovered in herds with a moderate or high SCC were less likely to be susceptible to the tested antimicrobials. The effect of lactation stage did not meet the defined significance level but was considered borderline significant (*P* = 0.058). The observed trend was that the odds of being susceptible were higher for isolates recovered in mid-lactation and lower for isolates recovered in late lactation, when compared with isolates that were identified in early lactation cows. Based on the variance components, clustering at the isolate level was greater than clustering at the herd level. As such, the correlation among susceptibilities within an isolate was greater (ICC = 0.26) than the correlation of susceptibilities between two different isolates (ICC = 0.06).

**Table 5:** **Final multilevel logistic regression model of the susceptibility^a^ of 192 environmental streptococci isolates from bovine milk tested against 3 antimicrobials**.

	β	SE(β)	OR	95%CI(OR)		*P* value	Overall *P* value
**Intercept**	1.43	0.55	–	–	–	–	
**Antimicrobial**							
Ampicillin	0.66	0.41	1.93	0.86	4.32	0.111	
Penicillin	−2.08	0.46	0.12	0.05	0.31	<0.001	
Tetracycline	Ref.	–	–	–	–	–	
**Species**							
*Streptococcus uberis*	Ref.	–	–	–	–	–	
*Streptococcus dysgalactiae*	−2.41	0.68	0.09	0.02	0.34	<0.001	
*Streptococcus* spp.^b^	−0.89	0.54	0.41	0.14	1.17	0.095	
*Lactococcus* spp.^c^	−0.05	0.58	0.95	0.31	2.94	0.925	
*Enterococcus* spp.^d^	−1.42	0.61	0.24	0.07	0.81	0.021	
**Antimicrobial × species**^e^							<0.001
**Herd somatic cell count**^f^							0.033
≤150,000 cells/mL	Ref.	–	–	–	–	–	
151,000–250,000 cells/mL	−1.01	0.52	0.36	0.13	1.01	0.052	
251,000–400,000 cells/mL	−1.52	0.59	0.22	0.07	0.69	0.010	
**Days in milk**^g^							0.058
0–100	Ref.	–	–	–	–	–	
101–200	0.68	0.38	1.97	0.94	4.14	0.072	
201+	−0.39	0.37	0.68	0.33	1.39	0.290	

**Variance**	**Estimate**	**SE**					
Herd level	0.27	0.25					
Isolate level	0.90	0.58					

*^a^Susceptible according to CLSI (20, 23)*.

*^b^Streptococcus spp. other than S. uberis, S. dysgalactiae, and S. agalactiae*.

*^c^L. lactis and L. garvieae*.

*^d^E. faecalis, E. saccharolyticus, E. faecium, E. pseudoavium, E. avium, E. casseliflavus, and E. hirae*.

*^e^Interaction term between antimicrobial and species*.

*^f^Herd somatic cell count = average within herd somatic cell count from 2007 to 2008*.

*^g^Days in milk at the time of milk sample collection*.

*OR, odds ratio*.

The breakdown of isolates among the three sample types was 44.8% (86/192) from apparently healthy lactating quarters, 30.2% (58/192) from post-clinical mastitis, and 25.0% (48/192) collected post-calving. The results of the logistic model of susceptibility considering sample type as the main independent variable are presented in Table 6. The odds of being susceptible was two times greater in isolates recovered in post-mastitis samples when compared with isolates recovered in non-clinical lactating quarters; there was no significant difference in susceptibility between isolates recovered in post-calving samples and isolates recovered in normal lactational samples. There was no interaction between sample type and antimicrobial, and compared to the baseline of tetracycline, the odds of susceptibility was significantly greater for all antimicrobials except for penicillin, which was not significantly different from tetracycline. As with the previous two models, correlation between the antimicrobial susceptibilities was greater within a single isolate (ICC = 0.23) than between two different isolates from the same herd (ICC = 0.04).

**Table 6:** **Final multilevel logistic regression model of the susceptibility^a^ of 192 environmental streptococci isolates from bovine milk tested against 8 antimicrobials with focus on the effect of sample type**.

	β	SE(β)	OR	95%CI (OR)		*P* value	Overall *P* value
**Intercept**	−0.33	0.24	–	–	–	–	
**Antimicrobial**							<0.001
Ampicillin	1.66	0.26	5.24	3.17	8.67	<0.001	
Ceftiofur	4.08	0.51	58.88	21.54	160.97	<0.001	
Cephalothin	3.72	0.45	41.15	17.01	99.55	<0.001	
Erythromycin	2.75	0.34	15.58	8.03	30.26	<0.001	
Penicillin	0.13	0.23	1.14	0.73	1.76	0.57	
Pen/novo	4.64	0.63	103.30	29.86	357.37	<0.001	
Pirlimycin	1.74	0.27	5.65	3.30	9.65	<0.001	
Tetracycline	Ref.	–	–	–	–	–	
**Sample type**							0.021
Lactational^b^	Ref.	–	–	–	–	–	
Post-mastitis^c^	0.73	0.26	2.07	1.24	3.46	0.006	
Post-calving^d^	0.26	0.27	1.29	0.77	2.18	0.33	

**Variance**	**Estimate**	**SE**					
Herd level	0.18	0.15					
Isolate level	0.77	0.26					

*^a^Susceptible according to CLSI (20, 23)*.

*^b^Isolates recovered in lactational and pre-dry-off milk samples*.

*^c^Isolates recovered in samples collected between 2 and 5 weeks after a recorded clinical mastitis event*.

*^d^Isolates recovered in samples collected between 0 and 14 days in milk*.

*OR, odds ratio*.

## Discussion

Determination of antimicrobial susceptibility of bovine mastitis pathogens *via* broth microdilution produces a quantitative result (the MIC) that can be used to compare data from various studies, either from different regions of the world or from the same region across different points in time. From a clinical perspective, classifying bacterial isolates as susceptible, intermediate, or resistant based on their MIC to a particular antimicrobial helps to guide treatment decisions. Clinical breakpoints for a particular antimicrobial are dependent upon the species of bacteria, the tissue or organ that is infected, and the host species. With regards to bovine mastitis pathogens, CLSI provides interpretive criteria for only three antimicrobials (ceftiofur, penicillin/novobiocin, and pirlimycin) against four or five pathogens [*S. aureus, S. agalactiae, S. dysgalactiae, S. uberis*, and *E. coli* (for ceftiofur only)]. Therefore, for other combinations of antimicrobial and pathogen, researchers and practitioners are required to use breakpoints derived from human data (20, 23). The appropriateness of applying these non-specific breakpoints is questionable but is, in most cases, the best available approach.

The results of this research support continued susceptibility of environmental streptococci to beta-lactam antimicrobials. This is in agreement with reports from other areas of North America, Europe, New Zealand, and South America (11, 12, 14, 15, 26–28). Beta-lactams are considered the first line of defense against environmental streptococci and are widely used in Canada. According to Saini et al. (3), regarding intramammary product usage in Canada, overall and, in the Maritime region specifically, penicillin + novobiocin was used most frequently, followed by penicillin, and first generation cephalosporins. Despite high usage rates of penicillin + novobiocin, 97.9% (188/192) of the isolates tested in this study had an MIC ≤ 0.5 μg/mL (or ≤1 μg/mL for *Enterococcus* spp.) that is below the ≤1 μg/mL cut point for susceptibility defined for *S. dysgalactiae* and *S. uberis*. Regarding cephalothin, the first generation cephalosporin included in this study, 95.8% (158/165) of the isolates were considered susceptible, and all species except for *Lactococcus* spp. had MIC_50_ and MIC_90_ ≤2 μg/mL, while the breakpoint for susceptibility for *Streptococci* spp. is set at ≤4 μg/mL.

A noteworthy departure from the expected susceptibility of environmental streptococci to beta-lactams was the apparent reduced susceptibility of *S. uberis* to penicillin and to a lesser degree, ampicillin. While the majority of studies from around the world report MIC_50_ of 0.03–0.06 μg/mL, MIC_90_ of 0.06–0.25 μg/mL, and susceptibilities ≥95% (12, 29–32), reduced susceptibility of *S. uberis* appears to be a sporadically occurring phenomenon with occasional reports in the literature (13). According to Rossitto et al. (33), in a study of 133 *S. uberis* isolates recovered from cases of bovine mastitis in California from 1997 to 1999, only 50.4% were considered susceptible, with an MIC_50_ and MIC_90_ of 0.25 μg/mL. More recently, in separate studies from New Zealand and Europe, both reported MIC_50_ of 0.03 μg/mL and MIC_90_ of 0.25 μg/mL with similar distribution of susceptibility to the current study, with 68.6 or 70.2% of isolates considered susceptible, 30.4 or 29.8% intermediate, and 1 or 0% resistant, respectively (14, 15). Finally, in the United States, a longitudinal study of antimicrobial susceptibility of mastitis pathogens detected a linear trend of decreasing susceptibility of *S. uberis* to penicillin; however, the within year range of susceptible isolates was much higher than in the current study, from 86.2 to 98.4% (34). The majority of isolates of *S. uberis* tested in the current study, classified as non-susceptible, had an MIC of only one or two dilutions above the intermediate susceptibility breakpoint. These findings support the results of Haenni et al. (13) who described a shift toward penicillin resistance among a subpopulation of *S. uberis* isolates and also identified the presence of resistance-associated mutations among isolates considered intermediately susceptible to penicillin. In the present study, four *S. uberis* isolates were considered resistant to penicillin with an MIC of ≥4.0 μg/mL; these isolates were also the outliers for ampicillin with MIC of 2–4 μg/mL. To the authors’ knowledge, MIC ≥ 4.0 against penicillin for *S. uberis* are very rarely reported in the literature. In a follow-up study, a subset of *S. uberis* and *S. dysgalactiae* isolates will be sequenced using next-generation sequencing to identify AMR genes and virulence factors, and to examine the relationship to the phenotypic expression of AMR.

Susceptibility to tetracycline was low for all species, and reduced susceptibility of environmental streptococci to tetracycline is frequently reported in the literature (12, 28, 30, 35, 36). Tetracyclines have been used extensively to treat many types of infections in both humans and animals for numerous years (37, 38). Many of the genetic determinants of tetracycline resistance have been shown to be actively transferred between bacterial genera and between hosts, both human and animal, and as a result, resistance to tetracycline is found in almost all bacterial genera (37). Furthermore, the persistence of tetracycline resistance genes in the apparent absence of selective pressure *via* exposure to treatment with tetracycline has been reported (37). Thus, although tetracycline has limited distribution in the udder and is not commonly used to treat intramammary infections (39), resistance to tetracycline among the isolates in the current study was not unexpected.

According to the results of the current study, there were differences in antimicrobial susceptibility between the genera and between *S. uberis* and *S. dysgalactiae*. This highlights the importance of obtaining the most detailed diagnosis possible when formulating treatment protocols. In the present study, isolates of *S. uberis* displayed lower susceptibility and higher MIC than isolates of *S. dysgalactiae*, with the exception of tetracycline. This has been reported by others (12, 32, 35) and is also reflected by the differences in treatment protocols recommended for these pathogens. While cure risk for *S. dysgalactiae* following a standard course of treatment is high, extended therapy is required to achieve similar cure risk for *S. uberis* infections (40–42).

Until recently, isolates of *Lactococcus* spp. and *Enterococcus* spp. were commonly identified as other streptococci (7), but, with the application of MALDI-ToF MS, accurate and easy speciation of numerous members of other environmental streptococci is now possible (43, 44). According to this study, important differences between susceptibility of *Lactococcus* spp. compared with the species of *Streptococcus* were highlighted, most notably higher MICs for pirlimycin and cephalothin. Pirlimycin is an analog to clindamycin, and clindamycin resistance has been proposed to be intrinsic in *L. gariveae* (45). In this study, all *L. gariveae* isolates (*n* = 16) had MIC > 2 μg/mL for pirlimycin, compared to *L. lactis* isolates of which 80% (12/15) had MIC ≤ 0.5 μg/mL and only two had an MIC > 2 μg/mL. The same species difference but against clindamycin have been reported by others (45–47), and similar results of *L. lactis* against pirlimycin have also been reported (7). For cephalothin, the MIC for *Lactococcus* spp. overall was higher than the other species, and a different MIC profile between *L. lactis* and *L. gariveae* was observed here as well and has been described by others (45–47). For *L. gariveae*, the MIC were all ≥4 μg/mL; for *L. lactis*, all but two outliers had an MIC ≤ 4 μg/mL, and the outliers had an MIC > 8 μg/mL. Lacking CLSI interpretive criteria for *Lactococcus* spp., we extrapolated breakpoints from the *Streptococcus* spp. This may have resulted in misclassifying isolates of *Lactococcus* spp. as susceptible or non-susceptible and thus affecting our interpretation of the results. This highlights the need for the development of additional bovine IMI-associated interpretive criteria for *Lactococcus* spp. and other bovine mastitis pathogens.

Intrinsic resistance of *Enterococcus* spp. to numerous antimicrobials is another example of how treatment failures might occur if causative pathogen diagnosis does not go beyond the group level. The MICs for *Enterococcus* spp. for ampicillin, penicillin, and tetracycline were higher than for the other species but were similar to those reported in data from other countries (29, 33, 48, 49). A clear bimodal distribution was observed toward tetracycline, which has been reported by others (33). This trend did not appear to be species-dependent as the species making up the genus group were present in both low and high MIC values. To a lesser degree, the *Enterococcus* spp. also appeared to have a bimodal MIC distribution for penicillin. Upon closer inspection, all isolates with MIC ≤ 0.125 μg/mL were *E. saccharolyticus*. These isolates also made up the group of *Enterococcus* spp. with an MIC ≤ 0.125 for ampicillin. To the authors’ knowledge, MIC data for *E. saccharolyticus* have, yet, to be reported. The majority of the remaining isolates of *Enterococcus* spp. had an MIC of 2 μg/mL for penicillin and 0.5–1 μg/mL for ampicillin.

According to the results of the logistic regression models, susceptibility was inversely related to herd level SCC such that isolates recovered in herds with moderate or high within-herd average SCC were less likely to be susceptible to the tested antimicrobials than isolates recovered in herds with low within-herd SCC. Considering that SCC is an indicator of udder health, it is logical to hypothesize that herds with a higher average SCC will have more IMI and will use antimicrobials more frequently than herds with a lower SCC. Exposure to antimicrobials is considered a driving force for AMR, thus herds using more antimicrobials could be expected to have reduced susceptibility among the bacteria isolated on the farm that on farms with low antimicrobial usage rates. A direct relationship between herd-level SCC and antimicrobial usage has not been supported by the current literature, but previous studies have reported that a more in-depth investigation in this area is required (3, 50). A second hypothesis explaining this association is the potential linkage between AMR and virulence (in this case, manifested as increased SCC). If the genetic determinants for pathogenicity and resistance are present on the same genetic element, or if a single genetic determinant confers both virulence and resistance, then both AMR and bacterial virulence will be coselected under the right selection pressures (51). To the authors’ knowledge, this phenomenon has not been explored among the streptococci associated with bovine IMI and requires further study.

Another interesting finding of the logistic models is that antimicrobial susceptibility was associated with stage of lactation of the host. Our results suggest that environmental streptococci isolated from cows in mid-lactation are more susceptible and conversely, isolates recovered in cows in late lactation are less susceptible when compared to the baseline of environmental streptococci identified in cows in early lactation. Blanket application of intramammary antimicrobials (dry cow therapy) at the end of lactation is common in North America and was employed as a mastitis control procedure in an estimated 88% of herds in 2007–2008 (52). Therefore, isolates recovered in the post-calving period were likely to have had recent exposure to antimicrobials. However, this study is neither able to determine if the IMI recovered post-calving were acquired in the dry period nor to determine if reduced susceptibility might have occurred as a result of either acquired resistance or elimination of a susceptible population by the dry cow therapy. Antimicrobial treatment in mid-lactation, at the time of peak milk production, is often disadvantageous from an economic perspective considering the cost of discarded milk due to antimicrobial residues (53). As a result, farmers might preferentially chose non-antimicrobial alternatives for the treatment of clinical mastitis (i.e., massage of the udder and frequent milking to flush out the infection) when cases arise during this high production stage of the lactation cycle. The prevalence of chronic IMI increases with increasing DIM (31), and this chronicity can be attributed in part to reduced susceptibility to antimicrobial treatment, thus it is not surprising to observe reduced susceptibility among the environmental streptococci recovered in late lactation.

Finally, according to the model including sample type, environmental streptococci isolated from post-clinical mastitis samples were more susceptible than isolates recovered from non-clinical lactating quarters. This apparent association is contrary to the principle that antimicrobial use propagates resistance and differs from Saini et al. (19), who concluded that there was no detectable difference in resistance proportion among isolates of *S. aureus, E. coli*, and *Klebsiella* spp. collected from intramammary infection, subclinical mastitis, and clinical mastitis cases. For the analysis of susceptibility, isolates originating from post-mastitis samples were preferentially chosen if more than one isolate of a species was recovered from an individual cow. It was our expectation that isolates recovered in milk samples collected post-mastitis would represent bacteria that failed to be eliminated in response to antimicrobial treatment and thus display reduced susceptibility. Unfortunately, animal-level treatment information was not available. The bacteria recovered in the samples collected at the time of mastitis diagnosis were not characterized by MALDI-ToF MS but rather were identified as *S. uberis, S. dysgalactiae*, and other *Streptococci* spp. based on traditional phenotypic and biochemical analysis. Based on this initial classification, in 50% (29/58) of cases had the same species (*S. uberis* or *S. dysgalactiae*) or group (other *Streptococci* spp.) identified at the time of diagnosis and in the subsequent sample. Without strain-typing of the pathogens causing the initial case of clinical mastitis and recovered in follow-up samples, it is impossible to determine if the isolates recovered in the period of 2–5 weeks after the onset of disease represented a persistent infection or a new intramammary infection. Based on the results of the current study, it is likely that, in cases treated with one of the commonly used beta-lactam antimicrobials, the infection was eliminated and the bacteria recovered post-mastitis represented a new infection.

In all three multilevel logistic models, the ICC at the isolate level was greater than at the herd level, indicating that most of the unexplained variation in antimicrobial susceptibility was within individual bacterial isolates (or cows). This suggests that the patterns of susceptibility were similar across all 18 farms (range: 54–92%; median = 81%) and thus susceptibility to antimicrobials among environmental pathogens is of importance to all farms experiencing environmental streptococcal intramammary infection. This also highlights the importance of obtaining the most detailed diagnosis possible, including both bacteriological culture and antimicrobial sensitivity testing, when formulating treatment protocols. Furthermore, interpretive criteria specific to bovine mastitis pathogens are scarce, and there is a need for the development of additional bovine IMI-associated interpretive criteria.

## Conclusion

The results of this study support continued susceptibility of environmental streptococci to beta-lactam antimicrobials. A noteworthy departure from the expected susceptibility of environmental streptococci to beta-lactams was the apparent reduced susceptibility of *S. uberis* to penicillin. According to the results of the current study, there were differences in antimicrobial susceptibility between the genera and between *S. uberis* and *S. dysgalactiae*; however, susceptibility to tetracycline was low for all species.

## Author Contributions

MC was the lead author of this manuscript and was responsible along with JS and MS for the realization of the project. The other authors contributed to the design of the study, analyses, and interpretation of study results. All authors were involved in reviewing the manuscript.

## Conflict of Interest Statement

The authors declare that the research was conducted in the absence of any commercial or financial relationships that could be construed as a potential conflict of interest. The reviewer BB and handling Editor declared their shared affiliation, and the handling Editor states that the process nevertheless met the standards of a fair and objective review.
